# The effectiveness of Balint group work on the quality of work life, resilience, and nurse–patient communication skills among psychiatric nurses: a randomized controlled trial

**DOI:** 10.3389/fpsyg.2024.1212200

**Published:** 2024-01-24

**Authors:** Negar Kiani Yousefzadeh, Mansoureh Kiani Dehkordi, Mohsen Vahedi, Ali Nazeri Astaneh, Fatemeh Sadat Bateni

**Affiliations:** ^1^School of Behavioral Sciences and Mental Health, Razi Educational and Therapeutic Center, University of Social Welfare and Rehabilitation Sciences, Tehran, Iran; ^2^Psychosis Research Center, University of Social Welfare and Rehabilitation Sciences, Tehran, Iran; ^3^Fellowship of Psychotherapy, Psychosis Research Center, University of Social Welfare and Rehabilitation Sciences, Tehran, Iran; ^4^Department of Biostatistics and Epidemiology, Substance Abuse and Dependence Research Center, University of Social Welfare and Rehabilitation Sciences, Tehran, Iran

**Keywords:** Balint group, quality of work life, psychiatric nurse, resilience, Iran

## Abstract

**Background:**

Balint group training has gained popularity in medical practices as an intervention designed to enhance the quality of life, well-being, and communication skills of healthcare practitioners. Psychiatric nurses, in particular, encounter distinct challenges and stressors inherent in their profession, necessitating the development and implementation of effective interventions to assist them in coping with the difficulties they experience. In this vein, the current study aimed to investigate the effectiveness of Balint group training on quality of work life, resilience, and nurse–patient communication skills among psychiatric nurses.

**Methods:**

Thirty psychiatric nurses from Razi Hospital in Tehran were recruited via the purposeful sampling method in 2022 and were randomly assigned to either the Balint group, consisting of eight weekly one-hour training sessions, or a control group. Participants completed the Walton Quality of Work Life Questionnaire, Connor-Davidson Resilience Scale, and Communication Skills Scale before and after the intervention. The data were analyzed using the Analysis of Covariance (ANCOVA).

**Results:**

The study found no significant differences between the Balint group and the control group in terms of quality of work life, resilience, and nurse–patient communication skills.

**Conclusion:**

Findings suggest that Balint group training was not an effective intervention for improving the well-being and communication skills of psychiatric nurses. However, the study highlights the need for further investigation into the potential factors that may explain the lack of significant gains and offers insights for future research in this area.

## Introduction

In light of the escalating influx of patients to hospitals, healthcare professionals, particularly nurses, find themselves grappling with substantial mental strain. The surge in workload has precipitated heightened stress levels, sleep disturbances, a pervasive sense of loss of control, and interpersonal conflicts. These challenges collectively contribute to imposing severe physical and psychological burdens on the dedicated healthcare workforce (Elhami Athar et al., 2020; Huang H. et al., 2020; Kwak et al., 2020). The nursing profession is acknowledged for its inherent physical and emotional demands, encompassing the provision of empathetic support and care to patients and their families facing diverse illnesses (Olofsson et al., 2003; Sherman, 2004). This demanding nature of the role has been correlated with specific adverse outcomes for nurses.

The quality of work life (QWL) stands out as a crucial concern for nurses, representing the intricate balance between their work experiences and personal and professional needs within the nursing domain. This delicate equilibrium significantly shapes nursing productivity and patient outcomes, thereby contributing to the fulfillment of organizational and hospital objectives (Fu et al., 2015). QWL, in this context, embraces key factors such as job security, job responsibilities, interactions with coworkers, salary considerations, and overall job satisfaction. These elements collectively define the holistic well-being of nurses in their professional environment (Nair, 2013). In the realm of psychiatric nursing, where professionals grapple with unique challenges and stressors inherent to their work, the comprehension and promotion of QWL take on paramount significance. Psychiatric nurses shoulder the responsibility of caring for individuals with mental health conditions, demanding elevated levels of emotional intelligence, resilience, and effective communication skills. Consequently, their QWL profoundly influences their capacity to deliver high-quality patient care while safeguarding their own well-being within a demanding work environment. Measuring QWL becomes pivotal in gauging the overall job satisfaction and well-being of psychiatric nurses, offering insights into areas that may benefit from improvement in the work environment. A nuanced examination of factors contributing to nurses’ QWL facilitates the development and implementation of targeted interventions. These interventions could help augment job satisfaction, fortify resilience, and enhance communication skills among psychiatric nurses, ultimately culminating in improved patient care outcomes (Mosadeghrad et al., 2011; Almalki et al., 2012). Indeed, research consistently indicates a substantial correlation between lower QWL levels among nurses and a cascade of adverse outcomes. These encompass a decline in the quality of care provided, heightened rates of absenteeism, increased workplace accidents, suboptimal professional conduct, an elevated risk of medical errors, job dissatisfaction, burnout, heightened job turnover, resignation, clinical depression, and tragically, an increased risk of suicide (Mosadeghrad et al., 2011; Nayeri et al., 2011; Sharps et al., 2016; Bagwell et al., 2017; Kavalieratos et al., 2017; Nazari et al., 2019).

The challenges stemming from lower QWL among psychiatric nurses may transcend individual well-being, casting a shadow over crucial elements of nursing practice. One such critical facet is the nurse–patient relationship, particularly significant in psychiatric settings (for a review, see Strandås and Bondas, 2018). The quality of the interaction between a nurse and a patient plays a pivotal role in influencing patient care and overall outcomes. A positive nurse–patient relationship is linked to heightened patient engagement, improved treatment results, and increased satisfaction among patients (Molin et al., 2016; Molina-Mula and Gallo-Estrada, 2020). This relationship is characterized by essential attributes such as compassion, connection, competence, communication, and presence. In the realm of healthcare, it serves as the cornerstone, providing the foundation for the delivery of quality care and the attainment of positive outcomes (Grossman, 2022). Consequently, a diminished quality of work life for nurses has the potential to impact the nurse–patient relationship, subsequently affecting the caliber of care and patient outcomes.

Despite the challenges inherent in healthcare environments, certain personality variables, such as higher levels of resilience, may contribute to nurses continuing to provide high-quality care. Resilience, defined as the potential to cope effectively and adapt positively to adverse situations, has been identified as a potential buffer against the negative impacts of stress on individuals (Hunter and Warren, 2013). However, it is important to note that resilience is a personality trait that can vary among individuals. Consequently, nurses with lower levels of resilience may be more vulnerable to the stressors experienced in healthcare settings. Given these considerations, it seems that enhancing resilience could be crucial for psychiatric nurses in coping with the demands of their work, maintaining their overall well-being, and increasing their ability to provide high-quality care to patients.

All in all, given the vital role of psychiatric nurses and the psychological challenges they face, it is crucial to implement interventions targeting improvements in their QWL, communication skills, and resilience levels. This proactive approach could help create a supportive environment, benefiting both healthcare professionals’ well-being and the quality of patient care. In recent years, interventions based on ‘Balint groups’ training have been widely used in medical practices (Van Roy et al., 2015; Huang H. et al., 2020). In the 1950s, Michael Balint introduced seminars for general practitioners (GPs) that were later called ‘Balint groups’ (Balint, 1955; Horder, 2001). In these groups, practitioners from different professionals present and discuss cases of difficult interactions with patients. Generally, BGs comprise 6–12 members and one or two leaders, which are held on a weekly to monthly basis over several years. During the sessions, practitioners present cases, and subsequently, group members provide comments and express their thoughts, ideas, and emotions. This process helps practitioners gain a deeper and more comprehensive knowledge of the problems they experienced, and makes them more capable of understanding their relationships with patients (Van Roy et al., 2014, 2015). Further, Balint group training improves the competency of GPs in patient encounters and helps them to endure their job and find enjoyment and challenge in their associations with patients (Airagnes et al., 2014; Van Roy et al., 2015; Huang H. et al., 2020).

Several studies have supported the effectiveness of the Balint group training among nurses and healthcare providers. For instance, Sekeres et al. (2003) assessed the impact of a Balint-like physician awareness group on hematology-oncology fellows’ attitudes and changes in these attitudes. Findings showed that the intervention enhanced fellows’ development as physicians. Likewise, Airagnes et al. (2014) indicated that Balint group training helped medical students to better handle challenging clinical situations such as those presented by borderline personalities. In the same vein, Huang H. et al. (2020) and Huang L. et al. (2020) showed that Balint group training resulted in significantly relieved burnout and improved quality of work life for intensive care unit (ICU) nurses. Additionally, Kiani Dehkordi et al. (2020) indicated that online Balint groups had significant positive influences on the Coronavirus anxiety and resilience scores of healthcare workers caring for COVID-19 patients in Iran. On the other hand, a study by Huang H. et al. (2020) and Huang L. et al. (2020) found that the Balint group training did not significantly influence the burnout and job satisfaction of resident physicians in their first year of residency at a comprehensive hospital in China. Likewise, a short-term Balint group did not significantly improve the communication ability and self-efficacy of pre-examination and triage nurses during COVID-19 (Yang et al., 2021). Finally, a recent study in Iran demonstrated that Balint group sessions significantly enhanced communication skills and empathy levels among psychiatry residents (Astaneh et al., 2023).

Still, to the best of our knowledge, there is currently no research investigating the impact of Balint group sessions on psychiatric nurses, despite the acknowledged stress and challenges inherent in their profession. As outlined earlier, psychiatric nursing is characterized by various stressors, including unfavorable work environments, low compensation, insufficient positive feedback, heavy workloads, limited resources, the stigma associated with the field, intricate interactions with professionals from other mental health disciplines, and demanding relationships with patients (Edwards et al., 2000; Paris and Hoge, 2010). To address this research gap, our study aims to assess the effectiveness of Balint group sessions in improving QWL, nurse–patient communication skills, and resilience levels among nurses working in a psychiatric hospital unit.

## Method

### Participants and procedure

**Figure 1:**
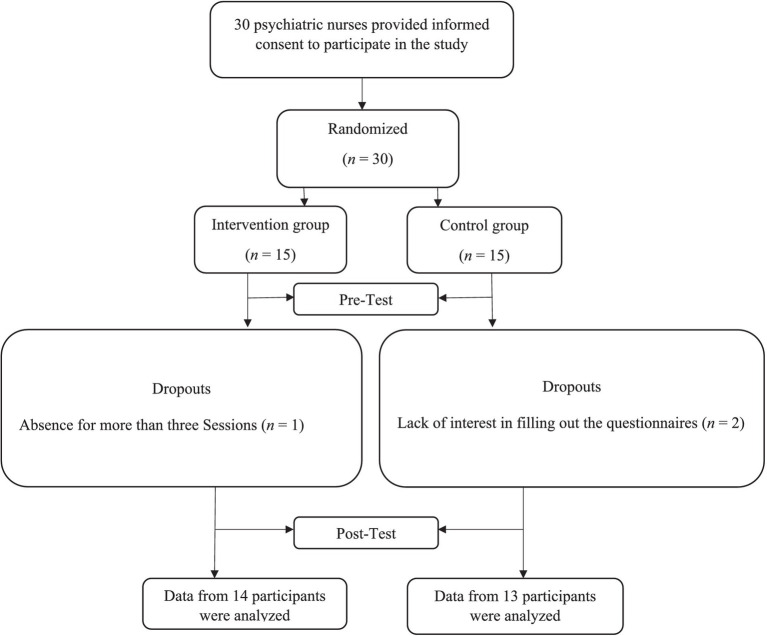
Process chart from recruitment to follow-up measurement.

For this study, we considered a two-group randomized controlled, pretest-posttest design. Participants included nurses in Razi Hospital in Tehran who were recruited in 2022 via the purposeful sampling method. Based on Browne’s (1995) proposed rule of thumb, we recruited 30 psychiatric nurses who were randomly assigned to either the intervention or control group (15 per group) using a simple randomization method via the rand function in Excel software (A graphic depiction of the recruitment process is presented in Figure 1). The enrollment and allocation of participants were conducted by a clinic specialist who was not involved in this research. This policy was designed to enhance transparency and minimize potential biases in the study’s design and implementation. The inclusion criteria consisted of (a) willingness to participate in the study and group meetings and providing the signed consent form; (b) lacking a history of the diagnosis of severe psychiatric disorder. Exclusion criteria included: (a) absence in at least two sessions; (b) lack of cooperation in completing the questionnaires.

First, an introductory interview (by the first and second authors who are trained in Balint group work) was conducted with participants in which they were explained about the aims of the study and ensured about the confidentiality of the data collected. Next, after obtaining a written consent form, the participants were asked to complete study measures and a questionnaire assessing demographic information, including age, gender, and marital status. Treatment sessions for the waiting list control group were conducted after the completion of the post-test phase. It is essential to highlight that throughout both the pre-test and post-test stages, assessments were carried out by clinic and research personnel who were deliberately kept blinded to participants’ group assignments. This meticulous blinding procedure was implemented to mitigate potential bias, ensuring that the assessments were conducted objectively and impartially.

Of the 30 participants in both groups, one and two participants dropped out from the intervention and control groups, respectively completing the 8-week intervention due to a lack of cooperation for more than 3 days. Therefore, data from 27 participants from intervention (*n* = 14) and control (*n* = 13) groups were analyzed (A graphic depiction of the recruitment process is presented in Figure 1). This study was first approved by the ethics committee of the University of Social Welfare and Rehabilitation Sciences (code number: IR.USWR.REC.1401.054). The study was also registered in the Iranian Registry of Clinical Trials (ID Number = IRCT20230211057383N1, Registration date: 2023-02-18). All participants provided informed consent after being explained about the study’s purpose and being assured about the confidentiality of the data.

### Intervention

Balint group training sessions consist of case reports and group discussions and attempts to throw light on the doctor-patient relationship. The detailed process of Balint group training is explained in Table 1. In this study, eight weekly group sessions (each session lasted for 60 min) were held for 2 months for the intervention group, while the control group did not receive any intervention within this period. The sessions were led by a trained leader and co-leader who had previously participated and trained in the Balint group work training course.

**Table 1:** The procedure of Balint group.

Step	Time (Minutes)	Content of the session
1	10	A psychiatric nurse presented cases that he/she carried in his/her mind for a long time.
2	10	Group members asked questions about the patient’s demographics and/or for completing the patient’s information. No emotional questions were asked.
3	25	The case presenter sit back from the group metaphorically and listen to the discussion and other members expressed their feelings and emotions about the cases
4	10	Rejoined the group and explained the new experiences of emotions, feelings, and thoughts about the case.
5	5	The leader and co-leader expressed thanks to all group members.

### Measures

#### Walton quality of work life questionnaire

The QWL consists of 35 items rated on a Likert type scale ranging from 1 (*“I am completely satisfied with”*) to 5 (*“completely dissatisfied”*). The components of Walton’s model of QWL are as follows: fair and adequate payment (four items), safety and health (six items), provision of growth and security opportunities (four items), legalism in the organization (four items), social relation of working life (five items), general living space (three items), the social cohesion of working life (four items), and development of human capabilities (five items). The lowest score is 35 and the maximum is 175. The scores are interpreted in the ranges of low (35–58), moderate (18–59), and high (118<). The Cronbach’s alpha coefficient for this questionnaire was 0.93 in an Iranian sample (Bakhshi et al., 2019).

#### Connor-Davidson resilience scale

The Connor-Davidson resilience scale (CD-RISC) was developed as a measure of assessing resilience. It contains 25 items, which are rated on a five-point Likert scale and range from 0 (*“Not true at all”*) to 4 (*“True nearly all the time”*). The original factor structure study yielded a five-factor solution including Personal competence, high standards, and tenacity (eight items), Trust in one’s instincts, tolerance of negative affect, and strengthening effects of stress (seven items) Positive acceptance of change and secure relationships (five items), Control (three items), and Spiritual influences (two items). The originally proposed five-factor model of the CD-RISC was replicated with an Iranian sample and yielded good reliability and validity (Nooripour et al., 2022). In the current study, we used the total CD-RISC score for data analysis.

#### Communication skills scale

The communication skills scale (CSS) was developed by Vakili et al. (2012) as a measure of communication skills among health workers. It includes 30 items rated on a Likert scale ranging from 1 (*“Extremely weak”*) to 5 (*“Excellent”*). Items assess communication skills in six areas of active listening (five items), interpretation and clarification of the audience’s conversations (five items), speaking skills (five items), feedback skills (five items), encouragement and praise (five items), and asking questions (five items). Scores are summed to yield subscales and total scores, with higher scores indicating elevated levels of communication skills. The Cronbach’s alpha coefficient for this questionnaire was 0.91 in the original study. In the current study, we used the CSS total score for data analysis.

### Data analyses

We used SPSS 20 software for data entry and statistical analyses. The normality of the distribution for outcome measures was tested using the Kolmogorov–Smirnov test, and the results supported the normality of the data (*p >* 0.05). The Chi-square test and independent *t*-test were implemented to compare descriptive variables of the groups, including marital status, level of education, and age. We performed a series of analyses of covariance (ANCOVA) to determine whether the groups differed in their reported levels of resilience, quality of work life, and nurse–patient communication skills scores using baseline values as covariates.^1^ The following rules of thumb are used to interpret values for Partial eta squared: η_p_^2^ = 0.01 indicates a small effect; η_p_^2^ = 0.06 indicates a medium effect; η_p_^2^ = 0.14 indicates a large effect (e.g., Elhami Athar, 2023). It was decided beforehand that a *p* level of less than 0.05 would be accepted as indicating statistically significant results.

## Results

As shown in Table 2, groups did not differ significantly on demographic variables, including age, marital status, and level of education. Thus, the groups were matched in these variables. To examine if the Balint group training has resulted in significant differences in resilience, quality of work life, and nurse–patient communication skills (dependent variables) scores between the groups, three sets of ANCOVAs with baseline values as covariates were conducted. Results indicated that there were no significant differences between the two groups in terms of resilience [*F*(1, 24) = 2.52, *p* < 0.13, η_p_^2^ = 0.10], quality of work life [*F*(1, 24) = 0.19, *p* < 0.73, η_p_^2^ = 0.005], and nurse–patient communication skills [*F*(1, 24) = 2.12, *p* < 0.16, η_p_^2^ = 0.08]. Table 3 presents the means and standard deviations of these variables in the pre-test and post-test for each group.

**Table 2:** The comparison of demographic data between control and intervention groups.

	Groups			Comparison	
Variables	Intervention (*n* = 14)	Control (*n* = 13)	Total (*n* = 27)	*t/Χ^2^*	*p*
Age, Mean (*SD*)	36.29 (6.90)	35.54 (8.21)	35.91 (7.55)	0.26	0.79
Gender (%)				1.8	0.18
Male	9 (64.3)	5 (35.7)	14 (51.9)		
Female	5 (38.5)	8 (61.5)	13 (48.1)		
Marital status (%)				2.49	0.11
Married	1.07.01	4 (30.8)	5 (18.5)		
Unmarried	13 (92.9)	9 (69.2)	22 (81.5)		

**Table 3:** Means and standard deviations for study variables by assessment step and ANCOVA results.

		Descriptive statistics		ANCOVA		
Variable group		Pre-test	Post-test			
		Mean (*SD*)	Mean (*SD*)	*F*	*p*	*η_p_^2^*
Resilience	Intervention	72.71 (10.17)	76.64 (11.60)	2.52	0.13	0.10
	Control	64.08 (14.37)	65.55 (15.13)			
Quality of work life	Intervention	40.29 (14.61)	41.86 (13.33)	0.19	0.73	0.005
	Control	36.83 (10.55)	33.58 (12.06)			
Nurse–patient communication skills	Intervention	83.50 (19.69)	89.00 (8.83)	2.12	0.16	0.08
	Control	78.33 (21.43)	86.58 (14.62)			

SD, standard deviation.

## Discussion

Balint group training sessions have provided promising results in helping health practitioners in dealing with the difficulties they experience in healthcare centers (Sekeres et al., 2003; Airagnes et al., 2014; Huang H. et al., 2020). To the best of our knowledge, this is the first study of examine the effectiveness of Balint group training sessions on psychiatric nurses’ QWL, nurse–patient communication skills, and resilience levels. In contrast to most prior studies (Sekeres et al., 2003; Airagnes et al., 2014; Huang H. et al., 2020; Kiani Dehkordi et al., 2020), our results showed that the Balint group training intervention did not significantly enhance the psychiatric nurses’ QWL, nurse–patient communication skills, and resilience levels.

Several factors may contribute to the observed pattern of results. To begin with, psychiatric nursing, as elucidated earlier, is inherently demanding and stressful, exerting a negative impact on the overall quality of life and job satisfaction of nurses (Edwards et al., 2000). Furthermore, in contrast to their counterparts in other healthcare disciplines, psychiatric nurses contend with intricate and demanding interactions with patients, which could amplify their stress levels (Paris and Hoge, 2010). Consequently, it is plausible that the therapeutic benefits derived from Balint group training interventions may not be as pronounced for psychiatric nurses, given the elevated levels of job-related challenges they regularly encounter. Second, it is crucial to consider that the intervention sessions in our study were delivered during the COVID-19 pandemic. The pandemic has had profound effects on the mental health and well-being of healthcare practitioners, leading to heightened levels of anxiety, depression, insomnia, stress, and burnout. Simultaneously, individuals with mental health issues experienced significant psychiatric symptoms, including anxiety and depression, especially during the peak of the pandemic, potentially contributing to mental health nursing providers facing increased levels of burnout and stress (García-Iglesias et al., 2020; Hao et al., 2020; Kameg et al., 2021). Given this context, the added strain imposed by the COVID-19 pandemic on psychiatric nurses may elucidate why the Balint group training intervention did not yield significant therapeutic gains for this specific group of healthcare professionals. Finally, the training sessions in our study spanned 8 weeks. Considering the exceptionally demanding nature of psychiatric nursing interactions with patients, it is conceivable that a more prolonged series of Balint group training sessions could potentially yield significant benefits. However, it’s noteworthy that the effectiveness of Balint group training has shown variability in different contexts. Notably, other studies have reported limited therapeutic gains. For example, a study involving first-year resident physicians in a comprehensive hospital in China found that Balint group sessions failed to significantly impact burnout and job satisfaction (Huang L. et al., 2020). Similarly, Yang et al. (2021) demonstrated that a short-term Balint group improved the communication ability and self-efficacy of pre-examination and triage nurses during COVID-19, but these improvements were not statistically significant. In the meantime, while our study is the first to explore the impact of Balint group training on psychiatric nurses, it is important to note that these results warrant replication, and definitive conclusions cannot be drawn solely from this single study.

Our results should be interpreted considering a few limitations. First, an interval of 2 months is a significantly short period to evaluate measurable changes in outcome variables, thus we recommend future studies include more sessions within a longer period to examine if the Balint group sessions could provide significant benefits for psychiatric nurses in the long run. Second, the current study was conducted during the COVID-19 pandemic, which adversely affected the mental health and overall well-being of healthcare practitioners, and this could have influenced the results of the current study. Third, a potential limitation of this study is the relatively small sample size of 30 psychiatric nurses. While this sample size was determined based on Browne’s proposed rule of thumb, further research with a larger sample size may be warranted to validate the results and enhance the generalizability of the findings. Finally, we did not conduct separate analyses across the gender groups. It is possible that Baling group training yield different pattern of results across males and females.

## Conclusion

As the first study to investigate the effectiveness of Balint group training intervention with psychiatric nurses, our findings indicate that the intervention did not result in significant improvements QWL, nurse–patient communication skills, and resilience levels among this group of healthcare practitioners. While this may be disappointing, our study provides valuable insights that could inform future research in this area. Despite the lack of significant findings, our study contributes to the growing body of literature on Balint group training in psychiatric nursing. We identified several possible factors that could explain the lack of significant therapeutic gains from the intervention. Our study underscores the need for further research in specific areas. Future investigations could explore the potential impact of extended Balint group training sessions on psychiatric nurses, examining whether a prolonged intervention period could yield significant therapeutic benefits. Also, comparative studies analyzing the effectiveness of Balint group training across diverse healthcare disciplines could offer valuable insights into contextual factors influencing outcomes. Moreover, studies with larger sample sizes could provide a more robust understanding of the generalizability and potential nuances of our findings. Finally, in future studies, an exploration of combined interventions or tailored adaptations of Balint group training may offer valuable insights into their effectiveness in addressing the distinctive needs and challenges inherent in psychiatric nursing practice. In conclusion, further investigation is warranted to better understand the effectiveness of Balint group training in improving the well-being and professional practice of psychiatric nurses, and ultimately enhancing patient care outcomes.

## Data availability statement

The raw data supporting the conclusions of this article will be made available by the authors, without undue reservation.

## Ethics statement

The studies involving humans were approved by the Ethics Committee of the University of Social Welfare and Rehabilitation Sciences. The studies were conducted in accordance with the local legislation and institutional requirements. The participants provided their written informed consent to participate in this study.

## Author contributions

NY: writing – original draft, software, and formal analysis. MD, MV, AA, and FB: writing – review & editing. All authors contributed to the article and approved the submitted version.

## Footnotes

^1^Although pre-tests showed no significant differences between the groups, we included them as covariates to control for any potential small differences that may have existed.
